# Andrologie becomes Basic and Clinical Andrology

**DOI:** 10.1186/2051-4190-23-1

**Published:** 2013-08-29

**Authors:** Roger Mieusset

**Affiliations:** CHU Hôpital Paule de Viguier, Toulouse, France

*Basic and Clinical Andrology (BaCA)* is a new scientific open access, international peer-reviewed journal covering all aspects of male reproductive and sexual health. *BaCA* has its roots in the journal formerly entitled *Andrologie* (Springer), the official journal of the French speaking Society of Andrology (SALF) for the past 25 years. *BaCA* is an evolution of this journal, adding an international dimension (publishing in English language) and is now the official journal of the SALF.

*Basic and Clinical Andrology* aims to bring to light the various clinical advancements and research developments attained in andrology around the world and thus help the field move forward. *BaCA* publishes many article types [1] in many areas, from basic research and clinical studies in animal models and humans related to male fertility, infertility, contraception as well as sexual and genital health.

The field of andrology cannot be simply reduced to spermatogenesis or spermatozoa, erection or erectile dysfunction. It also covers the development and maintenance of tissues, structures and functions at the molecular, cellular, tissue, organism levels to ensure male reproductive efficiency. As such, andrology should then be considered important enough in human life for knowledge not to be restricted to a small community.

As sexuality and genital health, as well as male fertility and contraception, are important parts of individual well being, accessibility to knowledge in andrology is required for any scientific or medical teams working in this area. In this way, an open access journal such as *Basic and Clinical Andrology* is a window onto the andrological world, with the potential to reach a much larger set of readers than any subscription-based journal, in print and online format. This ensures that author’s work will be disseminated to the widest possible audience and, hopefully, will offer better indexing opportunities [2, 3].

## The transition to open access

After a fruitful 4-year collaboration between *Andrologie* and Springer, the next logical step was to choose BioMed Central, which offers an open access publishing model on an online platform. Since January 2013, *Basic and Clinical Andrology* has received a growing number of manuscripts. The first three accepted articles are published today to mark the launch of the journal. However, we urge you to keep an eye on *BaCA* as there is more to come. In addition, you have the possibility to sign up for article alerts to be notified of the latest content.

The open access publishing model [4, 5] makes an article freely and universally accessible online, so an author’s work is available to readers at no cost and not limited by their library’s budget. Accessibility to knowledge in andrology is not only required by those who work in scientific and medical research but also to clinicians and biologists working with patients who may not necessarily be based in large institutions. In the open access publishing model authors retain the full copyright for their work and grant anyone the right to reproduce and disseminate the article, provided that it is correctly cited and no errors are introduced. The journal’s articles will be archived in PubMed Central [6], and other freely accessible full-text repositories.

## Financing open access

The traditional business model for scientific publishers relies on restricting access to published research, in order to recoup the costs of the publication process. This restriction of access to published research prevents full use of the results of this research, and is contrary to the interests of authors, funders and the scientific community as a whole [7]. The traditional subscription-based model is also becoming increasingly unsustainable; an increasing amount of research is being published whilst library budgets remain static.

In contrast, BioMed Central’s open access publishing model treats publication as the last phase of the research process. Article-processing charges (APCs) cover the cost of the publication process to allow free and immediate access to the research articles. APCs ensure transparency and allow publishers to compete to provide the best service at the best price. By coupling the cost of publication to research budgets, APCs ensure that the journal publishing system can scale to cope with an ever-increasing volume of research. The APC is a flat fee; there are no additional charges for colour figures, movies and large datasets.

## Finally, what happens when a manuscript is submitted to *BaCA*?

All manuscripts received by *BaCA* editorial office are subject to a fair peer-review process involving at least two reviewers selected from the international community with the help of a respectable and voluntary Editorial Board [8]. This peer review process lends validity to the resulting article and very often aids the researcher in publishing an improved article. This leads to less chance of errors and increased trust by the reader, with additive guaranties such as plagiarism detection and subscription to the principles of the Committee on Publication Ethics (COPE). After peer review, articles will be published online soon after editorial acceptance followed by indexing in PubMed.
